# Near Field Breast Tumor Detection Using Ultra-Narrow Band Probe with Machine Learning Techniques

**DOI:** 10.1038/s41598-018-31046-9

**Published:** 2018-08-22

**Authors:** Maged A. Aldhaeebi, Thamer S. Almoneef, Abdulbaset Ali, Zhao Ren, Omar M. Ramahi

**Affiliations:** 1 Department of Electrical Engineering, Hadhramout University, Al Mukalla, Yemen; 2 Department of Electrical and Computer Engineering, Prince Sattam University, Alkharj, Saudi Arabia; 3 Department of Electrical and Computer Engineering, Waterloo, N2L3G1 Canada

## Abstract

In this work, we propose a near-field microwave sensing modality that uses a single probe combined with a classification algorithm to help in detecting the presence of tumors in the human female breast. The concept employs a near-field resonant probe with an ultra-narrow frequency response. The resonant probe is highly sensitive to the changes in the electromagnetic properties of the breast tissues such that the presence of the tumor is gauged by determining the changes in the magnitude and phase response of the sensor’s reflection coefficient. A key feature of our proposed detection concept is the simultaneous sensing of tissue property changes to the two female breasts since the right and left healthy breasts are morphologically and materially identical. Once the probe response is recorded for both breasts, the Principle Component Analysis (PCA) method is employed to emphasize the difference in the probe responses. For validation of the concept, tumors embedded in a realistic breast phantoms were simulated. To provide additional confidence in the detection modality introduced here, experimental results of three different sizes of metallic spheres, mimicking tumors, inserted inside chicken and beef meat were detected using an electrically-small probe operating at 200 MHz. The results obtained from the numerical tests and experiments strongly suggest that the detection modality presented here might lead to an inexpensive and portable early and regular screening for breast tumor.

## Introduction

Breast cancer is one of the most common types of cancer among women and it is the second leading cause of death from cancer in women worldwide. In 2017, the American Cancer Society reported that more than 40,000 women will die from breast cancer in the US. In addition, it is expected that more than 250,000 new cases of invasive female breast cancer patients will be diagnosed in the US^1^. Detection of breast cancer tumors in their early stage (when they are small and have not spread), is critical for possible successful treatment^1^. X-ray mammography, magnetic resonance imaging (MRI), and ultrasound scanning are the most common clinical imaging modalities currently used for diagnosis and detection of breast cancer^2^. Screening x-ray mammogram for an abnormality detection requires further investigation to confirm if cancer is present because of a high false negative rate 0.2/1000 women screened at three yearly intervals, or a high false positive rate reaching 3.36% of all women screened in the UK^3^. In addition, it has been shown that the cumulative risk of the false-positive rate in women aged 50–69 years after 10 screening mammography rounds is 20–32% in Europe and 49– 63% in the United States^4^. Moreover, x-ray has other disadvantages such as ionization which poses a health risk, and compression of the breast tissues which causes significant discomfort to women^5^. Ultrasound uses high-frequency waves transmitted from a transducer through the breast tissues to form an image of the breast. Ultrasound provides a high contrast resolution; however, it lacks the spatial resolution of conventional mammography^6^. MRI is highly effective for imaging small abnormalities compared with mammography and ultrasound, and can be used effectively for women with dense breasts. However, MRI testing is very expensive and the technology is not widely available in most parts of the world^7^.

Microwaves imaging (MI) and detection modalities present an attractive alternative to the available clinical detection techniques for the primary reason that it requires inexpensive technology and is non-ionizing. The core principle behind all MI modalities is the contrast in the dielectric properties between normal and malignant breast tissues. Therefore, the significant differentiation in the dielectric properties of normal and malignant breast tissues can be used as the underlying principle for detection using electromagnetic waves. Research performed over the past 25 years confirms that malignant breast cancer tumors show sharp variation in their dielectric properties where the value of the permittivity and conductivity of the tumor tissues are higher than normal (healthy) tissues^8–12^. In the most comprehensive study of normal, benign and malignant breast tissue by Lazebnik *et al*.^9,13^, exhaustive measurements were performed to conclude that due to the complex network of glandular adipose and fibroconnective tissue in the breast, there is significant heterogeneity of dielectric properties in normal breast tissue.

MI can be classified into two categories: tomography^14,15^ and radar-based techniques^15,16^. Microwave tomography is based on illuminating the breast tissue to reconstruct the dielectric properties of the breast by using inverse scattering techniques that results in ill-posed nonlinear inverse problems^17^ which are computationally expensive and suffer from other limitations including resolution and accuracy^18–20^. The Dartmouth group has developed the first near-field microwave imaging system that illuminates the breast with 16 monopole antennas using a circular array operating in the frequency range of 300–1000 MHz^21^. Radar based modalities, on the other hand, are based on solving the forward scattering problem where the scattered signal from the breast is analysed to identify the presence of tumors^22^. An example of radar-based system was developed by Klemm *et al*.^23–25^.

In this work, we propose an electrically-small single element probe with an ultra-narrow frequency response^26^. The shift in the magnitude and phase of the reflection coefficient of the probe caused by the presence of a tumor existing inside a human breast is used as the primary detection technique. The primary objective behind our work is to provide an alternative detection technique that is not only reliable for early stage tumor detection but especially inexpensive, comfortable, non-ionizing and highly accessible to a wide sector of populations in different countries. Therefore, reliability of detection and low cost are two cornerstones of the new modality proposed in this work. We emphasize that the proposed detection technique focuses on identifying the presence of breast tumors rather than providing an image from which the presence of a tumor can be determined.

## Probe Design and Simulation

The probe used in this work is a dipole antenna small enough to confine most of the near-field radiation into the breast tissue to increase its sensitivity to variation in the dielectric properties of the tissues that are interrogated. Other types of antennas could be used such as loops or even patch antennas. However, we emphasize that the specificity of the probe topology is not of prime interest here but rather the entire system concept. Nevertheless, study of the sensitivity of different probes will be considered in a future separate study. For sufficient field penetration into the breast tissue, the operating frequency must be chosen carefully to ensure enough penetration level inside the dense breast. Without loss of generality, we select the frequency of operation to be 200 MHz which ensures sufficient field penetration in the human female breast.

**Figure 1:**
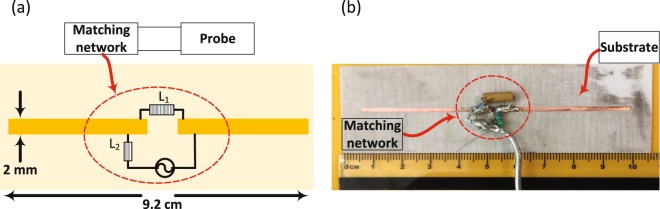
The printed electrically-small probe hosted on a dielectric substrate. (**a**) Schematic showing probe and the location of the matching network elements. (**b**) Photo of the fabricated probe and the matching network.

The probe was designed as a printed dipole of length 92 mm and trace width of 2 mm hosted on a RO4003 Rogers material with a thickness of 1.52 mm and a dielectric substrate of a relative permittivity of *ε*_*r*_ = 3.38 as shown in Fig. 1. The electrical length of the dipole is *λ*/12, (where *λ* is the wavelength in free space) which makes its radiation efficiency very low implying a flat, or near unity reflection coefficient (S_11_) response, which in turn, makes it highly insensitive to breast material changes. To enable the probe to be highly sensitive, the probe must be made an efficient radiator which lead to a highly defined ultra-narrow band-stop filter response by using a lossless matching network. The network was designed using the full-wave numerical simulation tool CST Microwave Studio^27^. The optimized matching network consisted of a series and parallel inductors having inductances of 0.36 μH and 0.49 μH, respectively with specific placement of the elements as shown in Fig. 1(a). We emphasize that both the values of the inductors and their location and orientation were optimized using CST.

**Figure 2:**
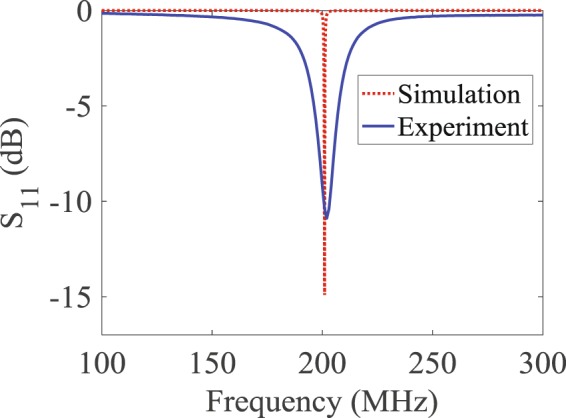
Response of the probe over the 100–300 MHz frequency band. The broadening of the bandwidth in the measurements arises form the dispersive nature of the material and circuit elements used in the construction of the probe.

The probe was fabricated and tested yielding strong agreement between the measurements and the simulations as can be observed from Fig. 2. Specifically, the agreement was very strong for the resonance frequency but we observe a deviation in the bandwidth which we attribute to non-ideal behavior of the elements and particularly the dispersive nature of all material involved in the fabrication of the probe (for instance, the inductance of real inductors is frequency dependent whereas the simulated ones have frequency independent inductance). The slight broadening of the bandwidth of the fabricated (real-world) probe in comparison with the ideal (numerical) probe has the advantage of providing additional features that enhance the detection discrimination when using the principle component analysis as will be shown below^16,28^.

The proposed probe was then simulated with a breast phantom to test its ability to detect the presence of tumours. In this work, we used the anonymous breast MRI datasets which were obtained from the University of Wisconsin online repository to build the breast phantom in the computer simulation technology (CST)^27,29,30^. The phantom is an anatomically realistic three dimensional (3D) numerical model with dielectric properties derived from the T1-weighted MRI images using a piecewise-linear mapping between MRI voxel intensity and the dielectric properties of the breast. The American College of Radiology (ACR) defines four categories of breast composition according to the radiographic density of breast fibrous and glandular tissues including class(I) almost entirely fat, class(II) scattered fibroglandular, class(III) heterogeneously dense, and class(IV) extremely dense^30,31^.

Without loss of generality, the model used in this work is the Heterogeneously Dense Breast ID: 062204” ACR classification: Class 3. The model has 0.5 × 0.5 × 0.5 resolution with 219 × 243 × 273 voxels. After processing the available data in Matlab, the data which includes the breast volume and the single Cole-Cole model for the frequency-dispersive tissues’ properties were used to construct a numerical model in CST as^32^:

1 $$\varepsilon (\omega )=\varepsilon ^{\prime} (\omega )-j\varepsilon ^{\prime\prime} (\omega )={\varepsilon }_{\infty }+\frac{{\rm{\Delta }}\varepsilon }{1+{(j\omega \tau )}^{1-\alpha }}+\frac{{\sigma }_{s}}{j\omega {\varepsilon }_{o}}$$

where *ω* is the angular frequency, *ε*′(*ω*) is the frequency-dependant dielectric constant, *ε*″(*ω*) is the frequency-dependant dielectric losses and *ε*_*o*_ is the free space permittivity. The *ε*_∞_, *σ*_*s*_, *τ* and *α* are the Cole-Cole model parameters obtained from the clinical experimental data. The breast model has the physical shape of a real human female breast. Moreover, with a high resolution of 0.5 mm, the model also includes the following eight tissue types: skin, muscle, glandular-1,2,3, and fat-1,2,3^31^.

**Figure 3:**
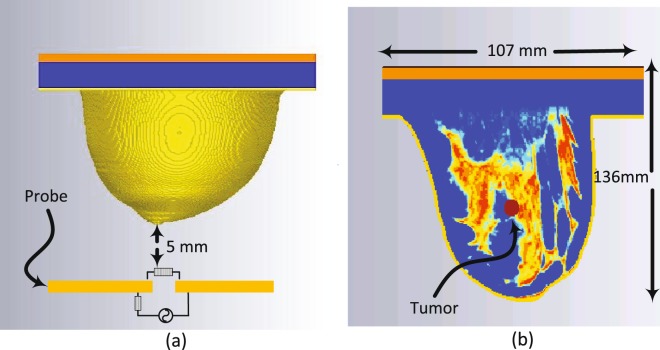
Simulation setup: (**a**) Ultra-narrow band probe with a stand-off distance of 5 mm from a 3D realistic numerical breast phantom model. (**b**) The embedded tumor in the numerical breast phantom.

The probe was calibrated with the numerical realistic breast phantom where the proposed probe is placed at a distance of 5 mm away from the healthy breast phantom as shown in Fig. 3(a). The magnitude and phase of the reflection coefficient of the probe were then recorded at 201 different frequencies spanning the range 100 to 300 MHz. Next, a tumor is inserted inside the same breast phantom with three different diameter sizes namely: 9 mm, 13 mm, and 17 mm, as shown in Fig. 3(b). The tumor’s dielectric properties were obtained from cancer surgery as documented in^9^. The magnitude and phase information were then recorded for the breast with the tumor. The data is then analysed with and without the tumor to decide whether or not a tumor is present.

The key aspect of the tumor detection modality introduced here is based on two findings. The first is the material and topological symmetry between the two healthy breasts of a woman^33,34^. The second is the unlikelihood that a woman would develop breast tumors in both breasts at the same time and the unlikelihood that a woman would develop identical breast tumors in both breasts at the same time^34^. The detection modality introduced here calls for the detection test to employ two identical probes positioned symmetrically with respect the two breasts. The responses of the two probes were then recorded (phase and magnitude of the reflection coefficients) and processed using the PCA method to decide whether or not a tumor is present. If the response of the probes from both breasts are identical, then the woman breasts are most likely free of tumors; otherwise, there is a likelihood of the presence of a tumor that can be either benign or cancerous. We fully realize that a discrepancy between the two does not conclusively determine which breast contains the tumor. Nevertheless, it is conceived that when such a device is realized, the outcome of such tests allows the healthcare provider to refer the women to additional screening using more expensive techniques if needed.

In the proposed detection method, the scattering parameters of the probe which contain the magnitude and phase features are extracted. Here, the feature vectors prior to applying the PCA analysis method are the magnitude and phase of reflection coefficient of the probe (the S11 or the scattering parameter of the probe). The feature vectors are then tabulated in a table containing 3 columns and 201 rows (not included in the paper for brevity). Each discrete value of the magnitude and phase changes with the frequency response. The magnitude and phase of the reflection coefficient of the probe were recorded at 201 different frequencies spanning the range 100 to 300 MHz. The two feature vectors: the magnitude and phase have dimensions of 201 rows and 2 columns. The first feature, the magnitude, is described by the first and second column which contains the frequency and magnitude points, respectively. The second feature, the phase, is described by the first and third column which comprises the frequency and phase points, respectively. The data was then entered into the PCA analysis method code to indicate the presence of a tumor inside a breast.

Next, we employ the PCA method to reduce the dimensionality of the problem by implementing a vector space transform^35^. The objective of PCA is to extract critical information from the scattering parameter response data set and to express this information as a set of orthogonal variables called principal components^36^. The eigen-decomposition of positive semi-definite matrices and the singular value decomposition of rectangular matrices are used for finding the principal components^36^. Thus, via mathematical projection, high dimensional original datasets can be reduced to small number of variables without losing much of the original information to analyze trends, patterns and outliers^35^.

**Figure 4:**
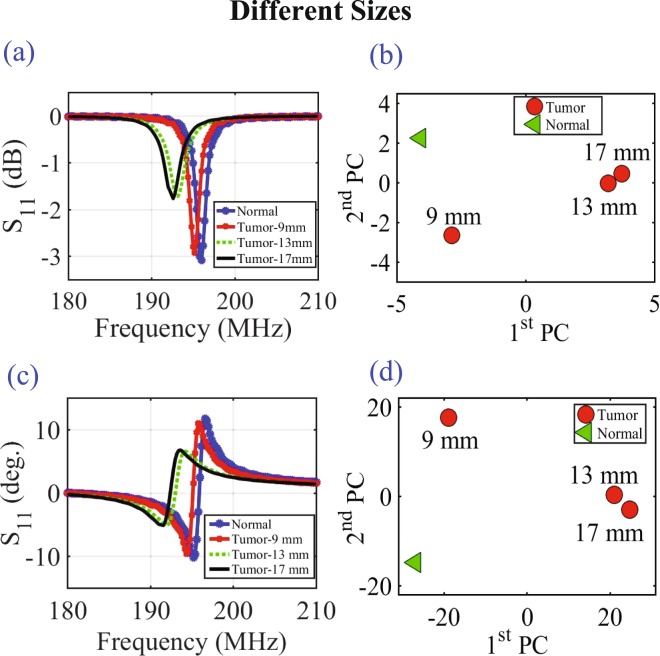
The probe response for normal breast tissue and breast tissue with different tumor sizes of 9 mm, 13 mm and 17 mm using the PCA feature extraction method. (**a**) The magnitude of *S*_11_, (**b**) the magnitude of *S*_11_ using PCA, (**c**)the phase of *S*_11_ and (**d**) the phase of *S*_11_ using PCA.

Once the probe response of the two probes are recorded (mimicking the scenario where a single probe is used for each of the two breasts), the PCA feature extraction method is applied for both the healthy and the tumourous cases. The results of Fig. 4(a,b) the magnitude of *S*_11_ using PCA, Fig. 4(c) the phase of *S*_11_ and Fig. 4(d) the phase of *S*_11_ using PCA show that the probe is capable of detecting breast tumors as small as 9 mm. Clearly the probe is more sensitive to larger tumor sizes as indicated by the more pronounced separation between the cases with and without tumors. We also observe that for the 9 mm tumor, the magnitude response was more effective than the phase response in the sense of providing higher discrimination.

## Experimental Validation

**Figure 5:**
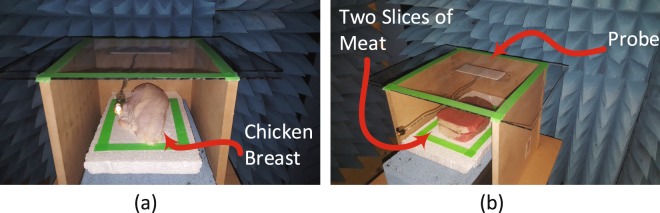
Experiment setup: (**a**) The ultra-narrow band probe with chicken breast. (**b**) The ultra-narrow band probe with two slice of meat.

In light of the preliminary and encouraging simulation results obtained above, an experiment was carried out to test the feasibility of the proposed concept in an environment that have resemblance to female breast tissues. The experiment setup consisted of the proposed electrically-small dipole probe, a keysight 8.5 GHz VNA, metallic spheres with different diameter sizes (mimicking a tumor), chicken breast tissue and a slice of a beef steak. The experiment apparatus was contained in a wooden box with an opening in one of the box sides to allow for easily placing and changing the position of the material under test inside the box as shown in Fig. 5. The experiment was performed inside an anechoic chamber to ensure that no energy is bouncing back from the surrounding environment and effecting the measured probe response.

**Figure 6:**
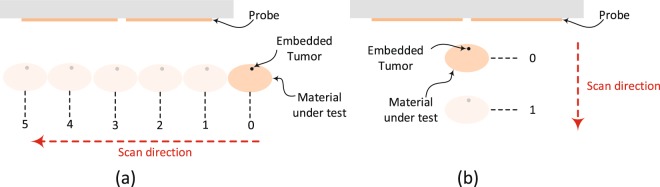
A schematic showing the experimental procedures. (**a**) Horizontal scan. (**b**) Vertical scan.

Initially, the material under test is scanned horizontally at 6 different positions as shown in Fig. 6(a). The main reason for performing such a scan is to determine the location where the probe sensitivity is highest (highest sensitivity refers to the highest shift experienced by the magnitude and phase of the S_11_ of the probe when compared to the original case). For instance, the S_11_ of the probe in the presence of an object placed at the edge of the dipole probe compared to that of the same object placed at the center of the probe is quite different. This is mainly due to the fact that a high electric field is confined within the gap of the dipole at the resonance frequency. When an object is placed in the vicinity of the gap, the probe’s response experiences higher shift in both the magnitude and phase than the case where the object is placed further away from the gap. Hence, the probe is highly sensitive to an object placed above or close to the gap. Once the position of highest sensitivity was found, (see Fig. 6(a)), the probe response was then recorded for this high sensitivity position. Although, the middle of the probe experienced the highest sensitivity, other locations away from the gap have a reasonable shift which can lead to a successful differentiation between the normal and the tumor breast tissue. Therefore, In a real-world detection scenario, the patient can position the probe in any location in front of the breast to perform the self examination.

**Figure 7:**
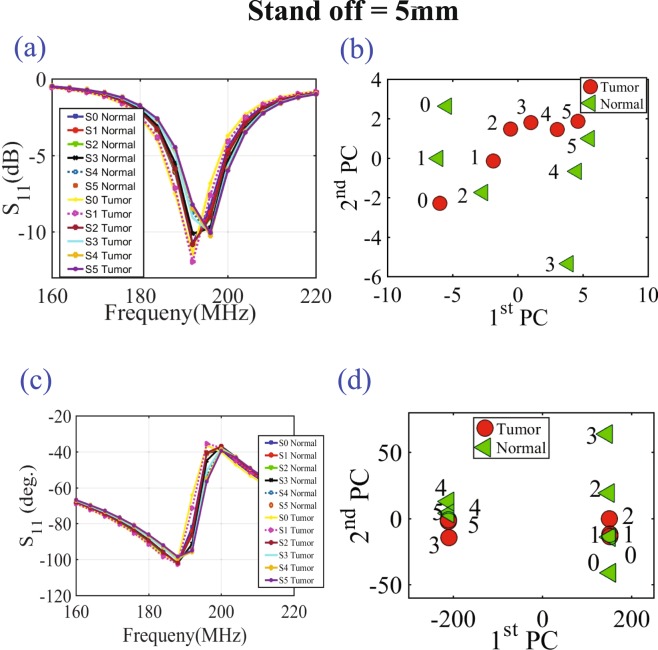
Experiment results of a chicken breast with an without an embedded metallic sphere of size 9 mm for all the horizontal scans (showing in Fig. 6(a)) and a stand-off distance of 5 mm (showing in Fig. 6(b)). The response of the probe was extracted and analyzed for (**a**) the magnitude of S_11_, (**b**) the magnitude of S_11_ using PCA, (**c**) the phase of S_11_ and (**d**) the phase of S_11_ using PCA.

**Figure 8:**
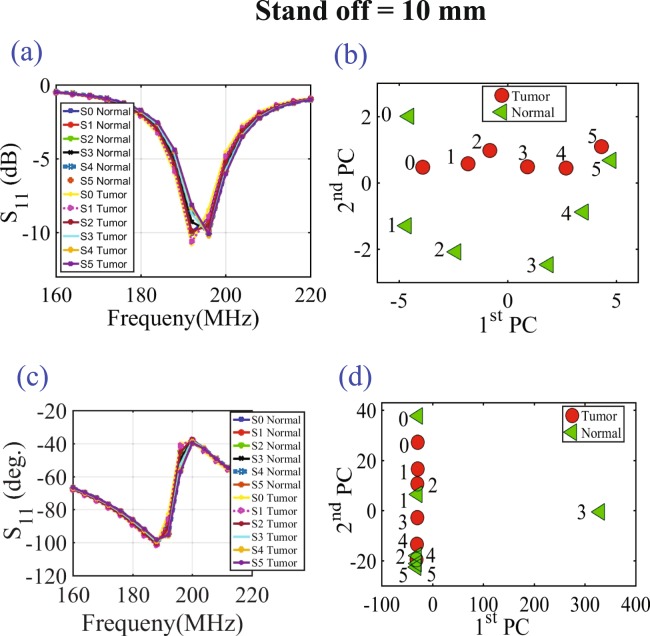
Experiment results of a chicken breast with an without an embedded metallic sphere of size 9 mm for all the horizontal scans (showing in Fig. 6(a)) and a stand-off distance of 10 mm (showing in Fig. 6(b)). The response of the probe was extracted and analyzed for (**a**) the magnitude of S_11_, (**b**) the magnitude of S_11_ using PCA, (**c**) the phase of S_11_ and (**d**) the phase of S_11_ using PCA.

Next, three different tests were performed: 1) with chicken breast, 2) a slice of beef, and 3) a hybrid of both. In each test, two main variables were changed. First, multiple metallic spheres, which will be embedded inside the material under test with different diameter sizes were used. Three different diameter sizes were used: 9 mm, 13 mm, and 17 mm to test the ability of the probe to detect various tumor sizes. For each tumor size, two different stand-off vertical distances were used as shown in Fig. 6(b). In the experiment, the scattering parameters seen from the port of the probe were recorded for both the phase and the magnitude. The phase and magnitude of the scattering parameters were analyzed with and without the PCA method for two vertical distances of 5 mm and 10 mm as shown in Fig. 6(b). The results are presented in Figs 7 and 8. It is evident from the results that the plots of the reflection coefficient of the probe in the presence of the metallic sphere for both the magnitude and phase as shown in Figs 7(a,c) and 8(a,c), experience a very slight shift that is hard to detect for the various locations labelled 0–5 (see Fig. 6(a)). Here, the difficulty in detecting the difference between the tumerous breast compared to a healthy one refer to the fact that a human eye cannot easily distinguish the change in either the magnitude or the phase of the probe. Additionally, by direct inspection of the magnitude and phase of the reflection coefficient, it is extremely difficult, if not impossible, to differentiate the healthy breast from the tumourous one, even if the process is automated, without additional classification procedure. However, when the data were plotted using the PCA method as shown in shown in Figs 7(b,d) and 8(b,d), a clear and obvious distinction is observed between the healthy breast tissue (no sphere) compared to that of the malignant case (with an embedded sphere).

**Figure 9:**
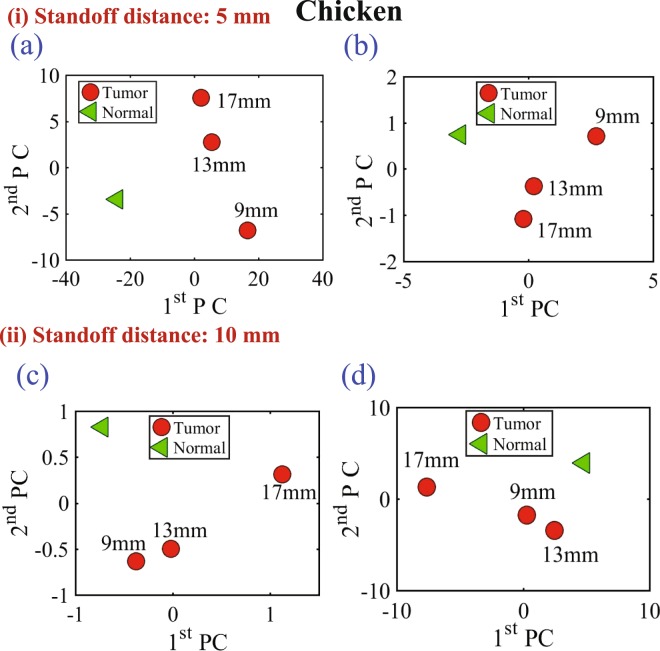
Experiment results of using three different sizes of metallic spheres of size 9 mm, 13 mm and 17 mm embedded in a the chicken breast using PCA with a stand-off distance of 5 mm for (**a**) magnitude of S_11_ and (**b**) the phase of S_11_) and stand-off distance of 10 mm for the (**c**) magnitude of S_11_ and (**d**) the phase of S_11_ using PCA.

**Figure 10:**
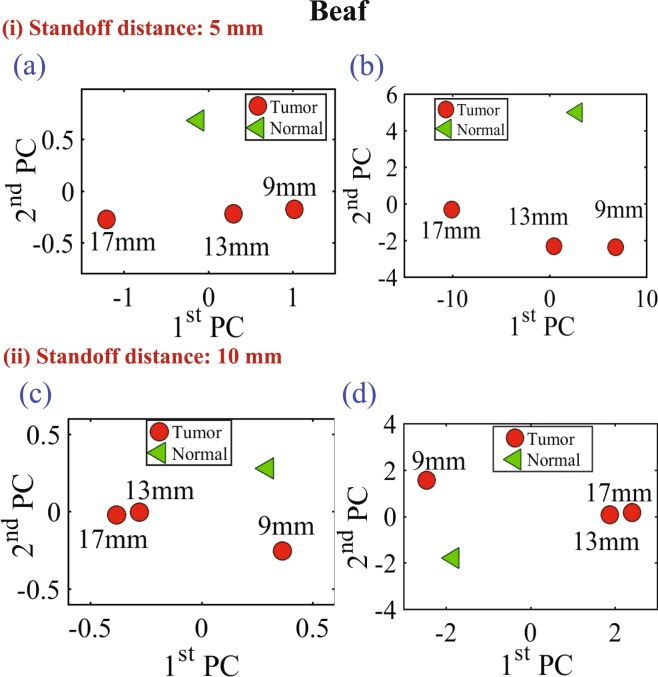
Experiment results of using three different sizes of metallic spheres of size 9 mm, 13 mm and 17 mm embedded in a slice of beef using PCA analyses with vertical distances of 5 mm (for the (**a**) magnitude of *S*_11_ and (**b**) phase of *S*_11_) and a vertical distance of 10 mm (for the (**c**) magnitude of *S*_11_ and (**d**) phase of *S*_11_).

**Figure 11:**
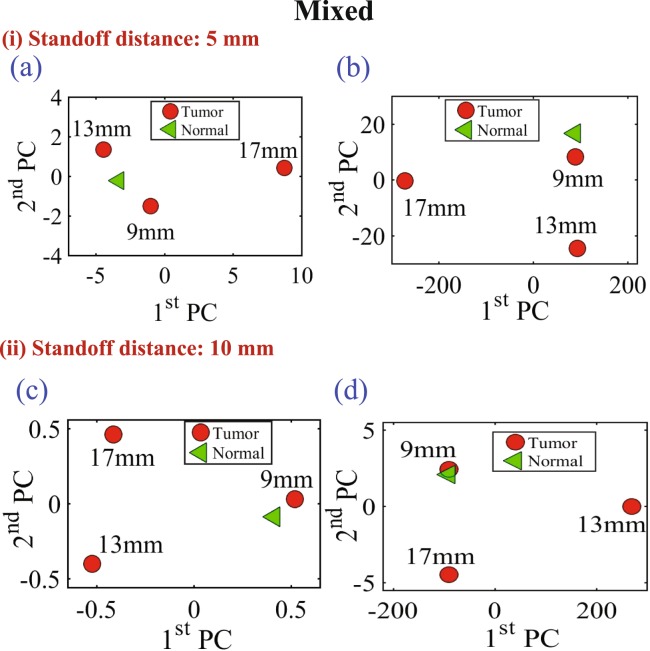
Experiment results of using three different sizes of metallic spheres of size 9 mm, 13 mm and 17 mm embedded in a mixture of a slice of beef and chicken breast using PCA analyses with vertical distances of 5 mm (for the (**a**) magnitude of *S*_11_ and (**b**) phase of *S*_11_) and a vertical distance of 10 mm (for the (**c**) magnitude of *S*_11_ and (**d**) phase of *S*_11_).

For the first experiment a chicken breast which includes skin, fat and meat was used as a testing material. The chicken was positioned at the fixed horizontal distance labeled 3 in Fig. 6(a). Then the magnitude and phase of the probe were extracted with two stand-off distance, namely 5 mm and 10 mm, as shown in Fig. 6(b). The experiment was then repeated after inserting a metallic sphere inside the same chicken breast. Please note that during the experiment, we labeled the location where the spheres were embedded inside the chicken to ensure a fair and identical comparison. The results obtained from this experiment is shown in Fig. 9. The second experiment was conducted with a slice of meat instead of chicken to test the strength of the probe to detect the variation inside the material with different electrical properties. Similarly, the test was done for different stand-off distances and different sphere sizes. The results obtained from this case is shown in Fig. 10. In the last experiment, a hybrid medium consisting of a chicken breast sandwiched between two slices of meat were used to test material with higher degree of heterogeneity than the previous two cases. The results are plotted in Fig. 11.

In all of the three experiments, the PCA analyzer provides a spatial separation between the normal and tumorous cases. In addition, it is noticeable that as the tumor size increases, the separation between the normal and the tumorous case increases, which indicates a more pronounced detection. Moreover, the separation between the cases with and without the tumor is more pronounced for the magnitude as compared to the phase of the S_11_ of the probe, which is similar to the behavior observed when interrogating the numerical breast phantom.

## Discussion

**Figure 12:**
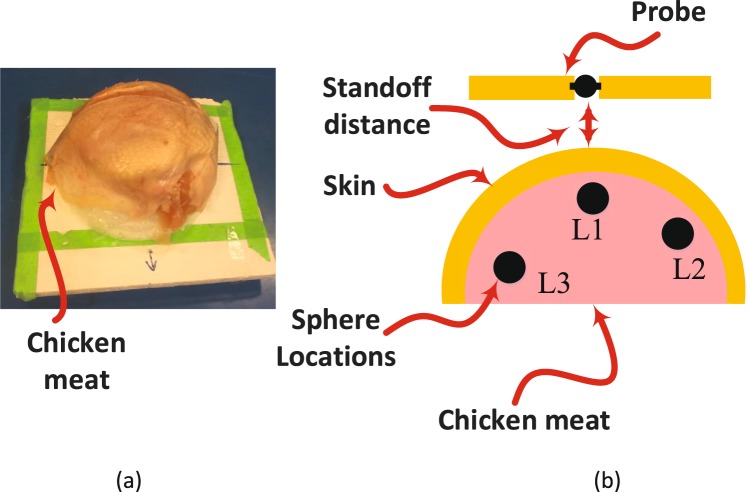
Experiment setup: (**a**) The chicken meat placed on hemispherical shape Styrofoam. (**b**) A schematic showing the inserted 9 mm sphere at three different location inside the chicken meat.

**Figure 13:**
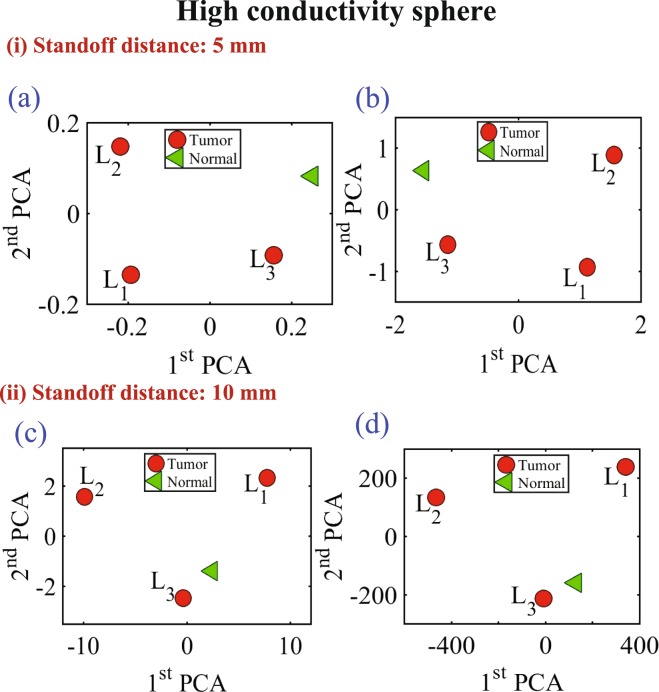
Experiment results of using a 9 mm metallic sphere embedded in a hemisphere chicken meat at three different locations using PCA analyses. (**a**) Magnitude of *S*_11_ and (**b**) phase of *S*_11_ at distance off 5 mm respectively. (**c**) Magnitude of *S*_11_ and (**d**) phase of *S*_11_ at distance off 10 mm respectively.

Since the realistic female breasts are of a hemispherical shape, is it critical to test the ability of the probe to detect tumors located at varies positions within a curved shape breast model. Therefore, an experiment was conducted consisting of a chicken meat placed on a Styrofoam of a hemispherical shape. Then a 9 mm metallic sphere was inserted in three locations consecutively inside the breast as shown in Fig. 12(b). The probe was then placed at a distance of 5 mm and 10 mm away from the breast. The experimental results are summarized in Fig. 13(i,ii) for a distance off of 5 mm and 10 mm respectively. Figure 13(a,b) show the magnitude and phase of *S*_11_ at distance off 5 mm respectively. Figure 13(c,d) show the magnitude and phase of *S*_11_ at distance off 10 mm respectively. It is evident from the results that there is a clear distinction in the magnitude and phase of the probe between the breast without tumor and the breast with tumor for all of the three locations. In the experiment, the locations of the tumors were selected to cover the outer peripheral and the middle parts of the breast. Such experiment proves the ability of the probe to detect tumors placed at different locations of a breast having a hemispherical shape regardless of the location of the tumor within the breast.

**Figure 14:**
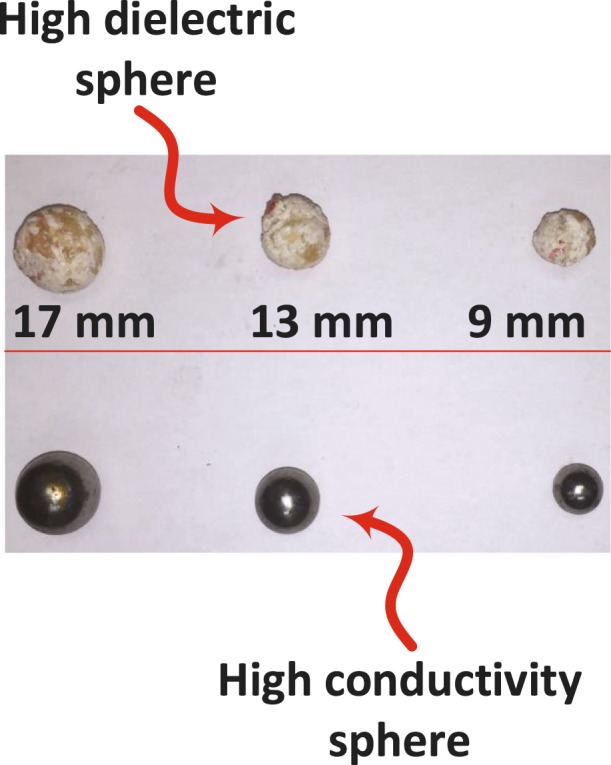
View of three different sizes of high conductive (metallic spheres) and high dielectric spheres (oil gelatine mixture spheres).

In all of the experiments performed thus far, we used metallic spheres to represent a tumor inside a breast. This is due to the fact that our main objective is to test the ability of the proposed probe to detect the dielectric variation within the breast. To study the ability of the probe to detect a realistic tumor which is not purely conductive, an experiment was conducted using tumors made of an oil and gelatine mixture as shown in Fig. 14. Such mixture is proven to yield dielectric properties similar to that of a realistic tumor^37–40^.

**Figure 15:**
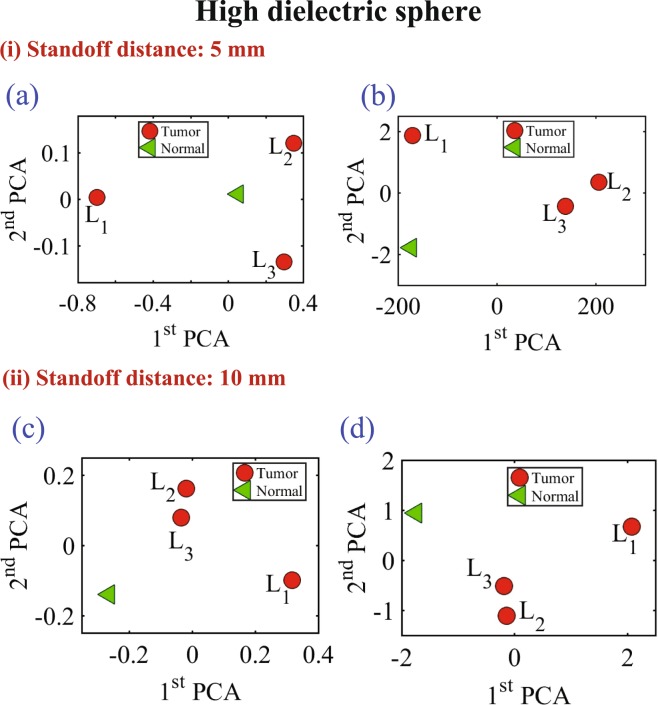
Experiment results of using a 9 mm high dielectric sphere embedded in a hemisphere chicken meat at three different locations using PCA analyses. (**a**) Magnitude of *S*_11_ and (**b**) phase of *S*_11_ at distance off 5 mm respectively. (**c**) Magnitude of *S*_11_ and (**d**) phase of *S*_11_ at distance off 10 mm respectively.

The experimental procedures used for the high conductivity sphere was repeated for the lossy dielectric spheres. We inserted a 9 mm high dielectric sphere at the same three different locations inside the hemisphere breast chicken. The probe response of the two testing without and with tumor at the different locations are recorded at standoff distance of 5 mm and 10 mm. Then the probe response was analyzed using the PCA as shown in as shown in Fig. 15. Figure 15(i,ii) show the magnitude and phase of S11 at distance off 5 mm and 10 mm respectively. Figure 15(a,b) show the magnitude and phase of *S*_11_ at stand off 5 mm respectively. Figure 15(c,d) show the magnitude and phase of *S*_11_ at stand off 10 mm respectively.

**Figure 16:**
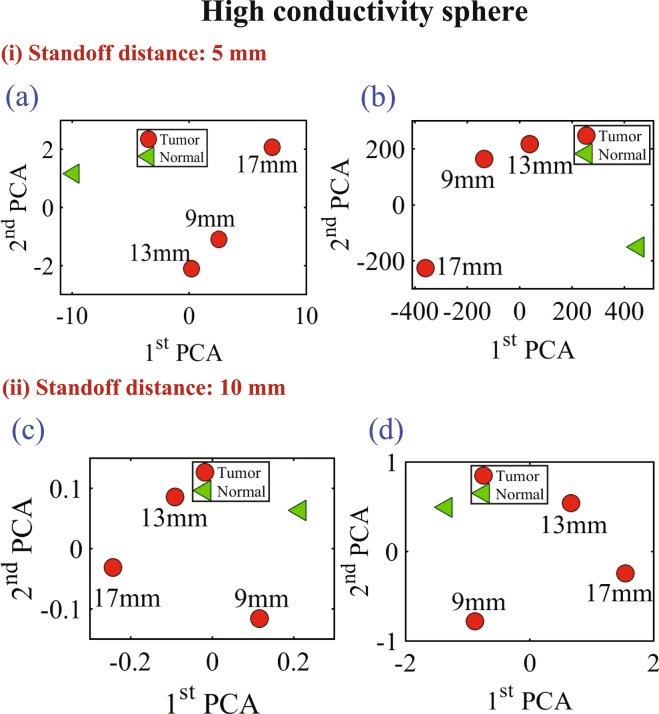
Experiment results of using three different sizes of high conductive spheres of size 9 mm, 13 mm and 17 mm embedded in a hemisphere chicken breast using PCA analyses. (**a**) magnitude of *S*_11_ and (**b**) phase of *S*_11_ at distance off 5 mm respectively. (**c**) Magnitude of *S*_11_ and (**d**) phase of *S*_11_ at distance off 10 mm respectively.

**Figure 17:**
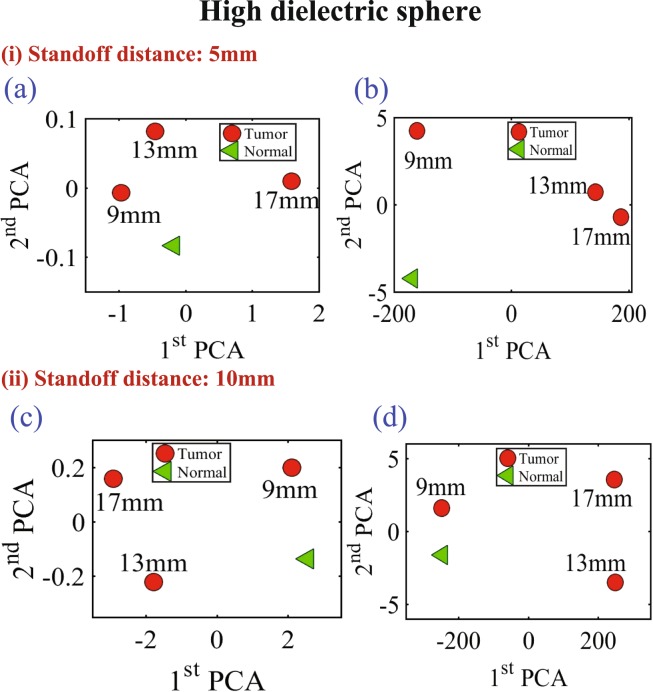
Experiment results of using three different sizes of high dielectric spheres (oil gelatine mixture spheres) of size 9 mm, 13 mm and 17 mm embedded in a hemisphere chicken breast using PCA analyses. (**a**) Magnitude of *S*_11_ and (**b**) phase of *S*_11_ at distance off 5 mm respectively. (**c**) magnitude of *S*_11_ and (**d**) phase of *S*_11_ at distance off 10 mm respectively.

In the last experiment, three different diameter sizes 9 mm, 13 mm and 17 mm of high conductive and high dielectric spheres were embedded inside the chicken meat. Two different measurements were conducted using spheres made of metal and the oil gelatine mixture. In both measurements, the probe was placed at two standoff distances, 5 mm and 10 mm, away from the phantom. The results are shown in Figs 16 and 17 for both the metallic based tumor and the oil-gelatine based tumor, respectively.

Figure 16(i,ii) show the magnitude and phase of *S*_11_ of the three high conductivity sphere sizes at standoff of 5 mm and 10 mm respectively. Figure 16(a,b) show the magnitude and phase of S11 at distance off 5 mm respectively. Figure 16(c,d) show the magnitude and phase of S11 at distance off 10 mm respectively. Figure 17(i,ii) show the magnitude and phase of *S*_11_ of the three high dielectric sphere sizes at standoff of 5 mm and 10 mm respectively. Figure 17(a,b) show the magnitude and phase of *S*_11_ of the three high dielectric tumor sizes at stand off 5 mm respectively. Figure 17(c,d) show the magnitude and phase of *S*_11_ at stand off 10 mm respectively. From the results, it is clear that the probe can detect the metallic based tumor and the oil-gelatine based tumor with different diameter sizes. From the results, it is clear that the probe can detect the metallic sphere and the oil-gelatine spheres with different diameter sizes.

## Conclusion

We presented an alternative microwave modality for breast tumor detection using a single probe for each of the two human female breasts. The sensing mechanism is simple and has the advantage of providing portability and comfort for the user. Our numerical results have demonstrated the ability of sensing a tumor that is as small as 9 mm buried inside breast tissues. In addition, experimental results show the high sensitivity of the probe which can detect the presence of a tumor inside a chicken breast having inhomogeneous dielectric variations. The feature extraction algorithm, the principle component analysis, was used to enhances the changes in the scattering parameters of the probe which aid in distinguishing between a healthy and tumorous breast.

The sensing modality conceived here is sharply different from other microwaves-based modalities that were based on reconstructing the dielectric properties profile of the breast. The modality presented here is fundamentally based on the complex interaction between the near-field of an electrically-small probe and the highly heterogeneous human female breast. The overall detection schemed is intended to provide women with regular initial stage breast tumor screening that is portable and comfortable. In this work, the feasibility of the concept was demonstrated by using human female breast phantoms and experimentally using chicken and beef tissues. Future work will focus on replacing the measurements’ devices with compact circuits to ensure full portability.

## Design Methodology

### Simulation Setup Method

The numerical simulations were conducted using the full-wave electromagnetic simulator CST Microwave Studio^27^. In the simulation setup, we created the realistic breast phantom and electrically-small probe using MATLAB^41^ and CST. The University of Wisconsin-Madison online repository provided a database of anatomically realistic numerical breast phantoms that can be used in CST. Each phantom has three ASCII text files including breastInfo.txt, mtype.txt, and pval.txt^42^. The breastInfo.txt file provides the basic information about the numerical phantom, such as breast ID: 062204, dimensions of the grid in units of grid cells [“s1”, “s2”, “s3”], and the classification of breast composition’ Heterogeneously Dense’. The mtype.txt file contains data of different voxel in the grid. The pval.txt file gives dielectric properties data for each voxel of the breast phantom^30^. In order to create a realistic breast phantom model in CST, three files were built as follows: (1) Vox file (ascii), (2) Material.txt file, and (3) Material property.txt or binary file. In the first step, we generated the material file and material property which contains all the data of the dielectric properties of all tissues of the breast using a single-pole Cole-Cole model in MATLAB. Then we created the vox file which contains the material properties. The final step involved importing the vox file into CST.

### Principal Component Analysis for Detection Classification

The PCA classification algorithm involves two steps: (1) Extraction the reflection coefficient of each recorded scan at the different stand-off distances and tumor sizes. (2) Using MATLAB which employed the PCA algorithm to show 2D plots that indicate whether or not a tumor is present.
